# Dietary Supplementation with Rumen-Derived *Streptococcus macedonicus* Improves Growth Performance and Carcass Characteristics of Barki Lambs

**DOI:** 10.3390/ani16182865

**Published:** 2026-09-11

**Authors:** Mahmoud M. Shaaban, Min Gao, Mohamed El-Sherbiny, Ahmed M. Abd El Tawab, Mostafa S. A. Khattab, Mohamed A. Radwan, Fatma I. Hadhoud, Yongbin Liu

**Affiliations:** 1 Biological Applications Department, Nuclear Research Center, Egyptian Atomic Energy Authority, Inshas, Cairo 13759, Egypt; 2 Inner Mongolia Academy of Agricultural & Animal Husbandry Sciences, Hohhot 010031, China; 3 Department of Dairy Science, National Research Centre, 33 Bohouth St., Dokki, Giza 12622, Egypt; 4 Department of Animal Production, Faculty of Agriculture, Cairo University, Giza 12613, Egypt; 5 Department of Animal Genetics, Breeding, and Reproduction, College of Animal Science, Inner Mongolia Agricultural University, Hohhot 010018, China; 6 State Key Laboratory of Reproductive Regulation & Breeding of Grassland Livestock, Inner Mongolia University, Hohhot 010021, China

**Keywords:** Barki lambs, carcass characteristics, direct-fed microbial, meat quality, nutrient digestibility, probiotic, rumen fermentation, *Streptococcus macedonicus*

## Abstract

This study evaluated a rumen-derived *Streptococcus macedonicus* isolate in Barki lambs using in vitro and in vivo approaches. The isolate was characterized for antibacterial activity and antimicrobial susceptibility. In vitro, a control diet was incubated for 24 h with 0, 0.002, 0.004, 0.008, or 0.016 g of *S. macedonicus*, and fermentation and digestibility were determined. In vivo, 40 Barki lambs were assigned to four dietary treatments for 210 d: control, control plus 1 g/d commercial probiotic, or control plus 1 or 2 g/d *S. macedonicus*. Feed intake, nutrient digestibility, growth performance, carcass traits, and meat quality were evaluated. The isolate showed antibacterial activity against *Escherichia coli*, *Pseudomonas aeruginosa*, and *Bacillus cereus*. In vitro, *S. macedonicus* increased dry matter digestibility (52.9% to 56.7%; *p* = 0.042) and total gas production (*p* < 0.0001). In vivo, dry matter digestibility increased from 66.3% to 75.2% (*p* = 0.002). Supplemented lambs had greater final body weight, average daily gain, and an improved feed conversion ratio (*p* < 0.001). Supplementation also improved carcass yield, dressing percentage, longissimus muscle area, lean proportion, water-holding capacity, and meat redness while reducing carcass fat percentage. Overall, rumen-derived *S. macedonicus* improved nutrient utilization, growth performance, and carcass and meat quality traits.

## Simple Summary

The use of probiotics is an increasingly attractive strategy for improving livestock productivity while reducing reliance on antibiotic growth promoters. In this study, we evaluated a strain of *Streptococcus macedonicus* isolated from the rumen of healthy sheep as a potential probiotic for growing Barki lambs. The isolate showed antimicrobial activity against several pathogenic bacteria and a predominantly susceptible antibiotic-response profile. Dietary supplementation with *S. macedonicus* improved nutrient digestibility and growth performance, resulting in greater body weight gain and better feed conversion efficiency than the control diet. It also increased carcass yield and longissimus muscle area, reduced carcass fat proportion, and improved meat quality traits, including water-holding capacity and redness. No significant treatment-related changes were detected in the measured blood biochemical variables. Both supplementation levels produced favorable responses, but increasing the dose from 1 to 2 g/day did not result in additional statistically significant improvements in the evaluated productive traits. These findings support further evaluation of rumen-derived *S. macedonicus* as a promising direct-fed microbial for improving the productivity and carcass quality of growing Barki lambs.

## 1. Introduction

Ruminants contribute substantially to food production by converting fibrous feeds and crop residues unsuitable for human consumption into high-quality animal products. This process depends on the rumen microbiota, which transforms dietary carbohydrates and proteins into volatile fatty acids, microbial protein, vitamins, and other metabolites required for maintenance and production. Inefficient ruminal fermentation can reduce nutrient utilization, feed efficiency, growth, and overall productivity. Accordingly, nutritional strategies that improve rumen function and microbial fermentation are increasingly important for efficient and sustainable sheep production, particularly where feed quality is limited [1,2,3,4].

Probiotics, or direct-fed microbials, have been widely investigated as nutritional tools to improve rumen fermentation, nutrient utilization, and productive performance. Their effects are strain- and context-dependent, varying with microbial species, dose, viability, diet, animal status, and management conditions [2,3,4]. In vitro and in vivo studies have shown that selected probiotic microorganisms can enhance substrate degradation, fermentation activity, nutrient digestibility, and animal performance, although responses are not uniform across studies [1,2,3,4,5,6]. Improvements in nutrient utilization may also translate into better growth, carcass development, and selected meat-quality traits; however, these responses likewise depend on the probiotic strain and production conditions [7,8,9,10,11,12,13,14]. These inconsistencies highlight the need to identify and evaluate new probiotic strains under relevant ruminant conditions.

Rumen-derived microorganisms may be particularly valuable as direct-fed microbial candidates because they originate from the ecological environment in which they are expected to function. Such isolates may possess physiological characteristics that favor adaptation and activity within the rumen. Among lactic acid bacteria, *Streptococcus macedonicus* has attracted attention because of its technological and probiotic-associated properties. The species belongs to the *Streptococcus bovis*/*Streptococcus equinus* complex and has been isolated mainly from traditional fermented dairy products [15]. Some strains produce macedocin, a lantibiotic with antimicrobial activity against several Gram-positive bacteria [16]. In addition, *S. macedonicus* ACA-DC 198 has undergone in vitro and in vivo safety assessment [17], while genomic and phenotypic studies have demonstrated substantial strain-dependent variation in gastrointestinal tolerance, adhesion, antimicrobial susceptibility, and putative virulence-associated traits [18]. These findings emphasize that the probiotic potential of *S. macedonicus* should be evaluated at the strain level.

Despite this interest, information on *S. macedonicus* in ruminant nutrition remains scarce. Most probiotic studies in sheep and cattle have focused on *Lactobacillus*, *Bacillus*, *Enterococcus*, yeast, and other established direct-fed microorganisms [2,3,4], whereas the functional potential of rumen-derived *S. macedonicus* has received little attention. In particular, studies integrating the isolation and characterization of a rumen-derived *S. macedonicus* strain with both in vitro ruminal fermentation assessment and in vivo evaluation of nutrient utilization, growth, and carcass responses are lacking.

Therefore, the present study isolated and characterized a rumen-derived *S. macedonicus* strain and evaluated its probiotic potential through antibacterial activity, antimicrobial susceptibility, and in vitro ruminal fermentation assays. Its effects on feed intake, nutrient digestibility, growth performance, feed efficiency, blood biochemical indices, carcass characteristics, and meat quality were subsequently investigated in growing Barki lambs. We hypothesized that supplementation with the rumen-derived *S. macedonicus* isolate would improve ruminal fermentation and nutrient utilization and thereby enhance productive performance and carcass characteristics without adversely affecting the measured blood biochemical variables.

## 2. Materials and Methods

### 2.1. Study Location and Ethical Approval

The in vivo experiment was conducted at the Animal Production Experimental Farm of the Nuclear Research Centre, Egyptian Atomic Energy Authority, Inshas, Egypt. Laboratory procedures, including the isolation and characterization of the probiotic strain, in vitro rumen fermentation, and chemical analyses, were performed at the Laboratory of Dairy Animal Production, National Research Centre, Dokki, Giza, Egypt.

All experimental procedures involving animals were performed in accordance with the institutional guidelines for the care and use of experimental animals and were approved by the Ethics Committee of the Nuclear Research Centre, Egypt (Approval No. 233/2024). Animal handling and management throughout the study complied with the approved ethical standards to ensure animal welfare.

### 2.2. Isolation and Characterization of Streptococcus macedonicus

#### 2.2.1. Isolation and Identification of *Streptococcus macedonicus*

Rumen fluid was collected from freshly slaughtered Barki sheep and transported anaerobically to the laboratory in a pre-warmed thermos flask. Anaerobic bacteria were isolated using serial dilution and enrichment techniques as previously described [19]. Briefly, aliquots of rumen fluid were inoculated onto selective media and incubated anaerobically at 39 °C for 24 h. Distinct white colonies were selected, transferred into de Man, Rogosa, and Sharpe (MRS) broth, and incubated under the same conditions.

Total genomic DNA was extracted from overnight bacterial cultures by alkaline lysis. The nearly complete 16S rRNA gene was amplified using the universal bacterial primers 27F (5′-AGA GTT TGA TCC TGG CTC AG-3′) and 1492R (5′-GGT TAC CTT GTT ACG ACT T-3′). PCR amplification was performed in a 25-µL reaction mixture containing 2.5 µL of 10× STR reaction buffer, 20 ng of total genomic DNA, 0.5 µM of each primer, and 1 U of Taq DNA polymerase. Amplification consisted of an initial denaturation at 94 °C for 5 min, followed by 35 cycles of denaturation at 94 °C for 1 min, annealing at 50 °C for 2 min, and extension at 72 °C for 2 min, with a final extension at 72 °C for 7 min.

PCR products were separated by electrophoresis on a 0.8% agarose gel at 75 V for 90 min. The gel was stained with ethidium bromide, and DNA bands were visualized under UV illumination at 254 nm. The amplified 16S rRNA gene product was purified and subjected to DNA sequencing by Applied Biotechnology Services (San Luis Obispo, CA, USA). The resulting sequence was compared and aligned with sequences available in the GenBank database using the Basic Local Alignment Search Tool (BLAST) of the National Center for Biotechnology Information (NCBI; https://www.ncbi.nlm.nih.gov/) [20]. Based on the 16S rRNA gene sequence analysis, the isolate was identified as Streptococcus macedonicus. The sequence was deposited in GenBank under accession number MW800768.

#### 2.2.2. Antibacterial Activity of the Isolated Strain

The antimicrobial activity of the isolates was evaluated using the agar well diffusion method [21]. Seven pathogenic indicator bacterial strains were obtained from stock cultures maintained in the Dairy Microbiology Laboratory at the National Research Centre: *Escherichia coli* O157:H7 (ATCC 6933), *Bacillus cereus* (ATCC 33018), *Salmonella typhimurium* (ATCC 14028), *Yersinia enterocolitica* (ATCC 27729), *Staphylococcus aureus* (ATCC 20231), *Pseudomonas aeruginosa* (ATCC 9027), and *Listeria monocytogenes* (ATCC 7644). Each strain was activated in tryptone soy broth through fermentation at 37 °C for 24 h. One milliliter of the culture of the activated indicator strain (104 cells mLG-1) was inoculated into 20 mL of Mueller-Hinton agar (Becton Dickinson, Franklin Lakes, NJ, USA) and poured into Petri dishes. Following agar solidification, wells with a diameter of 5 mm were excised from the agar using a sterile borer, and 50 µL of the isolate (*Streptococcus macedonicus*) was introduced into each well. The presence of an inhibition zone was considered indicative of antimicrobial activity, and the zone diameter was reported in millimeters. A diameter greater than 1 mm around the well was considered a positive result, with larger diameters indicating higher antimicrobial activity. The zone diameter of the wells cut in the nutrient agar medium was 5.0 mm, and the diameter of the inhibition zone (DIZ) of the negative control for each bacterium was also 5.0 mm. When the DIZ was 5.0 mm, the sample exhibited no inhibitory activity against the bacterium.

#### 2.2.3. Antimicrobial Susceptibility of the Isolated Strain

The antimicrobial susceptibility of the isolates was assessed following the method outlined by CLSI [22]. Antimicrobial discs were sourced from Oxoid (Oxoid, Hampshire, UK) with the following concentrations: AMP-10: ampicillin (10 µg), P-10: penicillin (10 U), VA-30: vancomycin (30 µg), CFR-30: cefadroxil (30 µg), E-15: erythromycin (15 µg), S-10: streptomycin (10 µg), K-30: kanamycin (30 µg), and TE-30: tetracycline (30 µg). Bacteria were classified as resistant to antibiotics if the clear zone diameter was ≤15 mm and as susceptible if the clear zone diameter was ≥21 mm.

### 2.3. In Vitro Evaluation of the Rumen-Derived Streptococcus macedonicus

#### 2.3.1. Source of the Rumen Inoculum

Rumen inoculum was collected immediately after slaughter from three cattle at the El-Munib slaughterhouse, Giza, Egypt. Before slaughter, the animals were fed a diet consisting of concentrate feed mixture and berseem hay (50:50, DM basis) with free access to water, followed by a 12-h fasting period. Rumen contents were collected from the dorsal, ventral, and central regions of each rumen, combined in equal proportions, and filtered through four layers of cheesecloth into a pre-warmed Schott Duran bottle (Schott North America Inc., Elmsford, NY, USA). The rumen fluid was transported immediately to the Laboratory of Dairy Animal Production at the National Research Centre, under anaerobic conditions, while maintaining a temperature of approximately 39 °C. Upon arrival, the inoculum was continuously maintained at 39 °C in a water bath, thoroughly mixed, and diluted with the incubation buffer before use.

#### 2.3.2. Experimental Design and Incubation Procedure

The in vitro batch culture incubation was performed according to El-Sherbiny et al. [23] with minor modifications. The ingredient composition and chemical composition of the basal substrate are presented in Table 1. The same basal diet was subsequently used in the in vivo feeding trial. Individual feed ingredients were dried, ground separately, and thoroughly mixed on a dry matter basis to obtain a homogeneous substrate.

Approximately 400 mg of the substrate was weighed into ANKOM F57 filter bags (ANKOM Technology, Macedon, NY, USA), which were then transferred into 125-mL glass incubation bottles. Five experimental treatments were evaluated: (1) Control (basal substrate without probiotic supplementation); (2) SM1 (Control + 0.002 g *Streptococcus macedonicus*); (3) SM2 (Control + 0.004 g *S. macedonicus*); (4) SM3 (Control + 0.008 g *S. macedonicus*); and (5) SM4 (Control + 0.016 g *S. macedonicus*). All incubation bottles were pre-warmed to 39 °C before inoculation.

Buffered rumen inoculum was prepared by mixing rumen fluid with the incubation buffer at a ratio of 1:4 (*v*/*v*). The incubation buffer contained (per liter): 292 mg K_2_HPO_4_·3H_2_O, 240 mg KH_2_PO_4_, 480 mg (NH_4_)_2_SO_4_, 480 mg NaCl, 100 mg MgSO_4_·7H_2_O, 64 mg CaCl_2_·2H_2_O, 4 mg Na_2_CO_3_, and 600 mg cysteine hydrochloride. Subsequently, 40 mL of buffered inoculum was added to each incubation bottle.

Each treatment was represented by three incubation bottles within each run, in addition to three blank bottles containing buffered inoculum without substrate. The entire experiment was repeated on three independent occasions using freshly collected rumen fluid for each incubation run. Before incubation, all bottles were flushed with CO_2_, sealed with rubber stoppers and aluminum crimps, and incubated at 39 °C for 24 h in a shaking incubator operating at 100 rpm under anaerobic conditions.

#### 2.3.3. Sampling and Analytical Procedures

At the end of the 24-h incubation period, total gas production (TGP) was measured immediately using a calibrated glass syringe. Methane (CH_4_) concentration in the headspace gas was subsequently determined using the portable gas analyzer (Crowcon Detection Instruments Ltd., Oxfordshire, UK) described by El-Sherbiny et al. [23]. Methane production was expressed as both total CH_4_ production (mL) and CH_4_ production per gram of incubated dry matter (mL/g DM).

Following gas measurements, the incubation bottles were opened, and the pH of the fermentation medium was measured immediately using a calibrated digital pH meter. The incubation contents were then filtered through the pre-weighed ANKOM F57 filter bags to recover the undegraded substrate. The filter bags were thoroughly washed with distilled water, dried at 105 °C to constant weight, and used to determine in vitro dry matter digestibility (IVDMD).

The filtrate was collected for the determination of ammonia nitrogen (NH_3_-N) concentration according to Khattab et al. [19]. Values obtained from blank bottles were used to correct gas production and digestibility measurements where appropriate.

### 2.4. In Vivo Animal Experiment

#### 2.4.1. Animals, Diets, and Experimental Design

A total of 40 healthy growing Barki lambs (24 males and 16 females) with an average initial body weight of 26.5 ± 1.5 kg and an age of 120 ± 6 d were used in the experiment. Before the start of the trial, the lambs were stratified according to sex, age, and initial body weight and randomly allocated to one of four dietary treatments in a completely randomized design. The feeding trial lasted for 30 weeks (210 d).

Lambs were housed individually in 1.5 m^2^ pens with free access to fresh drinking water throughout the experimental period. The experimental diets were formulated to meet or exceed the nutrient requirements of growing lambs according to NRC [24]. Feed was offered twice daily at 06:00 and 17:00 h, and the daily feed allowance was adjusted to allow approximately 10% refusals for the determination of voluntary feed intake.

The basal diet consisted of 600 g/kg concentrate feed mixture and 400 g/kg clover hay on a DM basis. The ingredient and chemical compositions of the basal diet are presented in Table 1. The dietary treatments were as follows: (1) Control, basal diet without probiotic supplementation; (2) CPro, Control diet supplemented with 1 g/d of a commercial probiotic; (3) SMD1, Control diet supplemented with 1 g/d of *Streptococcus macedonicus*; and (4) SMD2, Control diet supplemented with 2 g/d of *S. macedonicus*. To ensure complete consumption of the probiotic, the appropriate daily dose was thoroughly mixed with a small portion of the morning concentrate and offered before the remaining ration.

#### 2.4.2. Growth Performance and Feed Intake

Lambs were weighed individually before the morning feeding at the beginning of the experiment and thereafter at two-week intervals throughout the feeding period. Body weight data were used to calculate total body weight gain (BWG) and average daily gain (ADG).

Feed intake was determined daily by recording the amounts of feed offered and refused for each animal. Dry matter intake (DMI) was calculated accordingly, and feed conversion ratio (FCR) was expressed as grams of DMI per gram of ADG. Animals were observed daily throughout the experiment, and no clinical abnormalities associated with the dietary treatments were detected.

#### 2.4.3. Digestibility Trials

The first 4 weeks of the feeding trial served as an adaptation period, during which the lambs were acclimated to the experimental diets, housing, and management conditions. Three nutrient digestibility assessments were subsequently conducted during the 10th, 20th, and 30th weeks of the experiment. Feed intake was recorded daily for each lamb by weighing the amounts of feed offered and refusals from the previous day.

During each digestibility assessment, representative samples of the experimental diets and feed refusals were collected. Fecal grab samples were obtained individually from each lamb twice daily at 07:00 and 18:00 h for three consecutive days. Feed, refusal, and fecal samples were dried in a forced-air oven at 60 °C for 48 h. Fecal samples were then pooled by lamb within each digestibility assessment. The pooled fecal samples, together with representative diet and refusal samples, were ground to pass through a 1-mm screen using a Wiley mill and stored until chemical analysis.

Acid-insoluble ash was used as an internal indigestible marker to estimate apparent total-tract nutrient digestibility according to Van Keulen and Young [25]. Apparent digestibility coefficients of DM, OM, CP, EE, NDF, and ADF were estimated from the concentrations of acid-insoluble ash in the diet and feces. Chemical analyses of the experimental diets, refusals, and fecal samples were performed as described in Section 2.5.

#### 2.4.4. Blood Sampling and Serum Biochemical Analyses

Blood samples (10 mL) were collected from the jugular vein of each lamb before the morning feeding during the 10th, 20th, and 30th weeks of the feeding trial. Samples were collected into plain vacuum tubes and centrifuged at 3000× *g* for 15 min to separate the serum. Serum was transferred into sterile microcentrifuge tubes and stored at −20 °C until analysis.

Serum concentrations of total protein, albumin, glucose, cholesterol, triglycerides, urea, aspartate aminotransferase (AST), and alanine aminotransferase (ALT) were determined using commercial diagnostic kits (Stanbio Laboratory, Boerne, TX, USA) according to the manufacturer’s instructions. Serum globulin concentration was calculated as the difference between total protein and albumin, whereas serum thyroxine (T4) concentration was determined using RIA-coated tube kits (RIAKEYTUBE II^®^, Goyang City, Republic of Korea).

#### 2.4.5. Slaughter Procedure, Carcass Evaluation, and Meat Quality

At the end of the feeding trial, four lambs were randomly selected from each treatment and slaughtered after an 8-h fasting period with free access to water. Live body weight was recorded immediately before slaughter. Following exsanguination, the head, hide, feet, and internal organs were removed according to standard commercial procedures. Dressing percentage was calculated as the ratio of hot carcass weight to pre-slaughter body weight.

Following dressing, linear carcass measurements were recorded directly on the dressed carcasses in centimeters using a flexible measuring tape and, where appropriate, a digital caliper. Measurements included carcass length, carcass height, carcass depth, paunch girth, round width, and leg circumference. After removal of the head, head length and head width were measured separately. All measurements were performed consistently by the same trained personnel to minimize measurement variability.

The left-side 9th–11th rib section was excised immediately after slaughter and weighed. The cross-sectional area of the longissimus dorsi muscle at the 9th rib was measured using a digital planimeter. Rib sections were chilled at 4 °C for 24 h and subsequently dissected into lean, fat, and bone to determine carcass composition. Subcutaneous fat thickness over the longissimus dorsi muscle between the 12th and 13th ribs was measured using a digital caliper [26].

Samples of the longissimus dorsi muscle were collected for subsequent determination of chemical composition and physicochemical characteristics, including color coordinates (L*, a*, and b*), water-holding capacity, cooking loss, and Warner–Bratzler shear force, as described in Section 2.5.

### 2.5. Chemical Analyses

Representative samples of the experimental diets, feed refusals, and feces were dried in a forced-air oven at 60 °C for 48 h, ground to pass through a 1-mm screen using a Wiley mill (Thomas Scientific, Swedesboro, NJ, USA), and stored for subsequent analyses. Dry matter (DM), ash, crude protein (CP), ether extract (EE), and crude fiber (CF) were determined according to the standard methods of the Association of Official Analytical Chemists [27]. Organic matter (OM) was calculated by difference.

Neutral detergent fiber (NDF) and acid detergent fiber (ADF) were determined according to the procedures of Van Soest et al. [28] using heat-stable α-amylase and sodium sulfite for NDF analysis. Fiber fractions were expressed exclusive of residual ash. Acid-insoluble ash (AIA) was determined according to Van Keulen and Young [25] and was used as an internal marker to estimate the apparent total-tract digestibility coefficients of nutrients.

The chemical composition of the longissimus dorsi muscle was determined using a FoodScan™ Meat Analyzer (FOSS Analytical A/S, Hillerød, Denmark). Meat color was measured after 30 min of blooming using a Chroma Meter (CR-410, Konica Minolta, Osaka, Japan) according to the CIE L*, a*, and b* color system [29]. Chroma (C*) and hue angle (h°) were subsequently calculated as previously described [30,31]. Water-holding capacity was determined using the filter-paper press method [32], while cooking loss and Warner–Bratzler shear force were determined according to the procedures described by Honikel [33].

### 2.6. Statistical Analysis

Data were analyzed using SAS^®^ OnDemand for Academics (SAS Institute Inc., Cary, NC, USA, https://www.sas.com/en_us/software/on-demand-for-academics.html, accessed on 15 August 2024). Before analysis, data were examined for normality using the Shapiro–Wilk test and for homogeneity of variance using Levene’s test.

Data from the in vitro experiment were analyzed using a mixed model in which treatment was included as a fixed effect and experimental run as a random effect. The three independent incubation runs were considered experimental blocks.

For the in vivo experiment, variables measured repeatedly during the feeding trial, including feed intake, nutrient digestibility, average daily gain, feed conversion ratio, and blood biochemical parameters, were analyzed using the PROC MIXED procedure. The model included treatment, sampling period, and the treatment × sampling period interaction as fixed effects. Lamb nested within treatment was included as a random effect, with the sampling period specified as the repeated effect and lamb considered the experimental unit. Variables measured once per animal, including initial body weight, final body weight, and total weight gain, were analyzed with treatment as the fixed effect and lamb as the experimental unit. Carcass characteristics and meat-quality variables were analyzed similarly, with the individual slaughtered lamb considered the experimental unit.

When a significant treatment effect was detected, treatment means were compared using Duncan’s multiple range test [34]. Results are presented as least-squares means with the standard error of the mean (SEM). Statistical significance was declared at *p* < 0.05, whereas 0.05 ≤ *p* < 0.10 was considered a statistical trend.

## 3. Results

### 3.1. Antimicrobial Activity and Antibiotic Susceptibility

The antimicrobial activity and antibiotic susceptibility profile of the isolated *Streptococcus macedonicus* strain are presented in Table 2. The isolate exhibited inhibitory activity against *Escherichia coli*, *Pseudomonas aeruginosa*, and *Bacillus cereus*, whereas no inhibition was observed against *Salmonella typhimurium*, *Listeria monocytogenes*, *Yersinia enterocolitica*, or *Staphylococcus aureus*. In addition, the isolate was susceptible to ampicillin, penicillin, cefadroxil, erythromycin, and tetracycline, showed intermediate susceptibility to vancomycin, and was resistant to streptomycin and kanamycin. These characteristics supported the selection of the isolate for subsequent in vitro and in vivo evaluations.

### 3.2. In Vitro Fermentation Characteristics

The effects of *Streptococcus macedonicus* supplementation on in vitro fermentation characteristics are presented in Table 3. Supplementation with *S. macedonicus* significantly improved in vitro dry matter digestibility (IVDMD) compared with the control (*p* = 0.042), with the highest values observed in the SM2, SM3, and SM4 treatments. Total gas production was also markedly increased by all supplementation levels (*p* < 0.0001), indicating enhanced fermentation activity.

Methane production was affected by the level of *S. macedonicus* supplementation (*p* = 0.003). The lower supplementation levels (SM1–SM3) increased methane production compared with the control, whereas methane production in SM4 did not differ significantly from the control, either as total CH_4_ production or when expressed per gram of incubated dry matter. Ruminal pH and ammonia-N concentration were not significantly affected by the treatments.

### 3.3. Feed Intake and Nutrient Digestibility

The effects of dietary supplementation with *Streptococcus macedonicus* on feed intake and nutrient digestibility are presented in Table 4. Supplementation with either the commercial probiotic or *S. macedonicus* significantly increased concentrate, clover hay, and total dry matter intake compared with the control treatment, whereas no significant differences were observed among the supplemented groups. Feed intake was also affected by the sampling period, with no treatment × sampling period interaction detected.

Apparent nutrient digestibility was markedly improved by probiotic supplementation. Lambs receiving *S. macedonicus*, particularly the SMD1 treatment, exhibited the highest digestibility coefficients for DM, OM, CP, EE, NDF, and ADF. Digestibility values in the SMD2 group were generally comparable to those observed in SMD1, and both treatments outperformed the commercial probiotic and control groups. Significant effects of sampling period were observed for all digestibility variables, whereas the treatment × sampling period interaction was not significant.

### 3.4. Growth Performance

The effects of dietary supplementation with *Streptococcus macedonicus* on the growth performance of growing Barki lambs are presented in Table 5. Initial body weight did not differ among the experimental groups, indicating a comparable baseline before the commencement of the feeding trial. However, probiotic supplementation significantly improved growth performance throughout the experimental period. Lambs receiving *S. macedonicus* (SMD1 and SMD2) exhibited greater final body weight, total weight gain, and average daily gain than those in the control and commercial probiotic groups. No significant differences were detected between the two *S. macedonicus* supplementation levels for these variables.

Feed conversion ratio was also significantly improved by *S. macedonicus* supplementation (*p* < 0.001), with both SMD1 and SMD2 requiring less feed per unit of body weight gain than the control and commercial probiotic treatments. Average daily gain and feed conversion ratio were influenced by the sampling period, whereas no significant treatment × sampling period interactions were observed.

### 3.5. Blood Biochemical Parameters

The effects of dietary supplementation with *Streptococcus macedonicus* on blood biochemical parameters are presented in Table 6. Neither the commercial probiotic nor *S. macedonicus* supplementation affected serum concentrations of total protein, albumin, globulin, glucose, urea-N, cholesterol, triglycerides, or thyroxine (T_4_), nor the activities of glutamate-oxaloacetate transaminase (GOT) and glutamate-pyruvate transaminase (GPT). Likewise, sampling period and the treatment × sampling period interaction had no significant effects on any of the measured blood variables. Overall, no significant treatment-related changes were detected in the measured blood biochemical variables at weeks 10, 20, and 30 of the feeding trial.

### 3.6. Carcass Characteristics

The effects of dietary supplementation with *Streptococcus macedonicus* on carcass characteristics are presented in Table 7. Lambs supplemented with *S. macedonicus* (SMD1 and SMD2) exhibited greater live body weight, hot carcass weight, and dressing percentage than the control group (*p* < 0.01), with no significant differences between the two supplementation levels for live weight or carcass weight. In addition, *S. macedonicus* supplementation increased the *longissimus dorsi* muscle area and lean proportion, while reducing carcass fat thickness, fat percentage, and bone percentage compared with the control treatment (*p* < 0.05).

Carcass measurements were also positively influenced by *S. macedonicus* supplementation. Lambs in the SMD1 and SMD2 groups had greater carcass length, height, depth, paunch girth, round width, and leg circumference than those in the control and commercial probiotic groups (*p* < 0.01). In contrast, head length and head width were not affected by dietary treatment (*p* > 0.05). Overall, both *S. macedonicus* supplementation levels produced comparable improvements in carcass yield and conformation.

### 3.7. Meat Quality Characteristics

The effects of dietary supplementation with *Streptococcus macedonicus* on the chemical composition and physical characteristics of lamb meat are presented in Table 8. Dietary treatment had no significant effect on the chemical composition of the longissimus dorsi muscle, including moisture, crude protein, collagen, and fat contents (*p* > 0.05). Likewise, meat brightness (L*), yellowness (b*), chroma, and hue angle were not influenced by probiotic supplementation.

In contrast, major physical quality attributes were significantly improved by *S. macedonicus* supplementation. Lambs receiving SMD1 and SMD2 exhibited greater water-holding capacity and higher redness (a*) values than the control and commercial probiotic groups (*p* < 0.01), whereas no significant differences were observed between the two *S. macedonicus* supplementation levels. These findings indicate that supplementation with *S. macedonicus* enhanced selected meat quality characteristics without altering its chemical composition.

## 4. Discussion

### 4.1. Antimicrobial Activity and Probiotic Potential of Streptococcus macedonicus

The rumen-derived *Streptococcus macedonicus* isolate exhibited inhibitory activity against both Gram-positive (*Bacillus cereus*) and Gram-negative (*Escherichia coli* and *Pseudomonas aeruginosa*) bacteria while displaying a predominantly susceptible antibiotic profile. These characteristics are consistent with those expected of a promising probiotic candidate and provided the rationale for its subsequent evaluation in both the in vitro fermentation and in vivo feeding experiments.

Antimicrobial activity is considered one of the principal functional characteristics of probiotic microorganisms, contributing to the suppression of undesirable bacteria through the production of antimicrobial metabolites such as organic acids, hydrogen peroxide, and bacteriocins [35,36]. Among *S. macedonicus* strains, the production of the lantibiotic macedocin has been identified as a distinctive feature, with demonstrated bactericidal activity against several Gram-positive bacteria [37]. Although previous investigations have focused mainly on dairy isolates, the antimicrobial activity observed in the present rumen-derived isolate suggests that this species may also possess functional traits beneficial within the ruminant gastrointestinal environment.

Safety evaluation is another essential prerequisite for probiotic development. Current EFSA guidance recommends that bacterial strains intended for use as feed additives should be susceptible to clinically important antimicrobials or, where resistance is detected, that it should be demonstrated to be intrinsic rather than acquired [38,39]. The predominantly susceptible phenotype observed in the present study provides preliminary phenotypic information relevant to the safety assessment of the isolate but does not establish its overall safety. Further strain-level characterization, including genomic assessment of antimicrobial resistance and other safety-related determinants, is required before practical application can be considered.

Overall, the antimicrobial activity, together with the favorable antibiotic susceptibility profile, indicates that the rumen-derived *S. macedonicus* isolate possesses functional characteristics consistent with a probiotic candidate, justifying its further evaluation in ruminant nutrition.

### 4.2. Effects of Streptococcus macedonicus on In Vitro Rumen Fermentation

Supplementation with the rumen-derived *S. macedonicus* isolate improved in vitro dry matter degradability and total gas production without significantly affecting ruminal pH or ammonia-N concentration. These responses indicate increased fermentation activity and substrate degradation under the conditions of the present in vitro experiment.

Improved substrate degradability following probiotic supplementation has been reported in previous in vitro studies, where probiotic microorganisms may enhance microbial fermentation and nutrient utilization through modulation of ruminal microbial activity [40,41,42]. Although microbial community composition was not evaluated in the present study, the increased dry matter degradability observed following *S. macedonicus* supplementation is consistent with an enhancement of substrate utilization.

Methane production showed a dose-dependent variation rather than a consistent reduction. The lower supplementation levels (SM1–SM3) resulted in greater methane production than the control, whereas methane production at the highest supplementation level (SM4) was statistically comparable with the control, both on a total-volume basis and when expressed per gram of dry matter. Therefore, the present results do not provide evidence that *S. macedonicus* supplementation mitigated methane production.

Ruminal pH and ammonia-N concentration remained unaffected despite the improvements in dry matter degradability and total gas production, suggesting that the observed fermentation response occurred without marked changes in these fermentation variables.

### 4.3. Effects of Streptococcus macedonicus on Feed Intake, Nutrient Digestibility, and Growth Performance

Dietary supplementation with the rumen-derived *S. macedonicus* improved dry matter intake, nutrient digestibility, final body weight, average daily gain, and feed conversion ratio compared with the control treatment. Although no significant differences were detected between the 1 and 2 g/day supplementation levels for several productive responses, this lack of statistical difference should not be interpreted as evidence of biological equivalence or as establishing an optimal supplementation dose. Rather, increasing the supplementation level from 1 to 2 g/day did not produce additional statistically significant improvements in the evaluated productive traits under the conditions of the present study.

The improvements in nutrient digestibility are consistent with the enhanced in vitro fermentation observed in the present study and indicate that the beneficial effects of *S. macedonicus* on ruminal fermentation translated into improved nutrient utilization in vivo. Similar increases in apparent nutrient digestibility following probiotic supplementation have been reported in growing lambs and sheep, where improved ruminal microbial activity enhanced fiber degradation and nutrient availability [43,44,45]. Although ruminal microbial populations were not determined in the present study, improved digestibility likely contributed to the greater nutrient supply available for growth.

Enhanced nutrient utilization was accompanied by increased feed intake and improved growth performance. Previous studies have similarly reported that probiotic supplementation increased voluntary feed intake, average daily gain, and feed efficiency in sheep, primarily through improved digestive efficiency and nutrient utilization [41,43,44,45]. The simultaneous improvement in feed conversion ratio observed in the present study indicates that the additional feed consumed was efficiently converted into body weight gain rather than simply increasing intake.

An important finding of the present study was the absence of additional productive responses at the higher supplementation level. Similar plateau responses have been described for other probiotic strains, suggesting that probiotic efficacy depends more on strain functionality than increasing dosage once an effective inclusion level has been achieved [44,45].

Overall, the improvements in feed intake, nutrient digestibility, growth rate, and feed efficiency support the beneficial effects of the rumen-derived *S. macedonicus* isolate on animal performance and are consistent with the enhanced fermentation characteristics observed during the in vitro evaluation.

### 4.4. Blood Biochemical Responses

Dietary supplementation with the rumen-derived *Streptococcus macedonicus* did not significantly affect the measured blood biochemical parameters compared with the control treatment. Thus, no treatment-related differences were detected in the evaluated serum biochemical indices at weeks 10, 20, and 30 of the feeding trial. Similar findings have been reported in sheep receiving probiotic supplementation, where blood biochemical variables were largely unaffected despite changes in productive performance [41,43,46]. However, the variables measured in the present study represent only a limited set of physiological indicators and should not be interpreted as a comprehensive assessment of the safety of the isolate. Further strain-level and longer-term safety evaluations are therefore warranted.

### 4.5. Carcass Characteristics and Meat Quality

Dietary supplementation with the rumen-derived *Streptococcus macedonicus* improved several carcass characteristics, including hot carcass weight, dressing percentage, Longissimus muscle area, lean proportion, and carcass dimensions, while reducing carcass fat thickness, carcass fat percentage, and bone proportion. In contrast, the chemical composition of the Longissimus muscle remained unchanged, whereas water-holding capacity and meat redness were significantly improved. These results demonstrate that the enhanced growth performance observed in probiotic-supplemented lambs translated into improved carcass yield and desirable meat quality characteristics.

The improvements in carcass weight and dressing percentage are consistent with the greater nutrient digestibility, feed efficiency, and average daily gain observed in the present study, indicating that the enhanced availability of nutrients promoted lean tissue accretion rather than excessive fat deposition. Similar improvements in carcass yield and muscle development have been reported in lambs receiving probiotic supplementation, although the magnitude of the response depends on the probiotic strain, dietary composition, and management conditions [43,44,45]. The greater Longissimus muscle area, together with the increased lean proportion observed in the present study, further supports the beneficial effects of the rumen-derived *S. macedonicus* on muscle development.

A notable finding was the reduction in carcass fat thickness and carcass fat percentage despite the higher slaughter weight. This response suggests improved nutrient partitioning toward lean tissue growth rather than fat deposition, thereby increasing the proportion of saleable meat. Similar responses have been reported in probiotic-fed lambs and goats, where improved ruminal fermentation and nutrient utilization enhanced carcass composition without compromising animal performance [43,44,47].

Although the chemical composition of the Longissimus muscle was unaffected, supplementation significantly improved water-holding capacity and meat redness. These attributes are among the most important determinants of fresh meat quality because greater water-holding capacity reduces moisture losses during storage and processing, whereas increased redness enhances consumer acceptance and perceived freshness. Similar improvements in meat color, water-holding capacity, tenderness, and antioxidant status have been reported following probiotic supplementation in lambs, particularly with lactic acid bacteria [12,48]. Because oxidative stability, muscle antioxidant activity, and myoglobin oxidation were not evaluated in the present study, the mechanisms responsible for these improvements cannot be confirmed. Nevertheless, the enhanced physical quality of the meat without changes in its proximate composition suggests that *S. macedonicus* primarily influenced postmortem meat characteristics rather than muscle nutrient composition.

Overall, supplementation with the rumen-derived *S. macedonicus* improved both carcass yield and selected meat quality attributes, complementing the improvements observed in nutrient utilization and growth performance. These findings indicate that this probiotic has potential not only to increase lamb productivity but also to enhance characteristics of commercial importance for the meat industry.

## 5. Conclusions

The present study demonstrated that dietary supplementation with a rumen-derived *Streptococcus macedonicus* isolate improved nutrient digestibility, feed intake, growth performance, feed efficiency, carcass characteristics, and selected meat quality attributes in growing Barki lambs. No significant treatment-related changes were detected in the measured blood biochemical variables at weeks 10, 20, and 30. Both supplementation levels produced favorable productive responses; however, increasing the supplementation level from 1 to 2 g/day did not result in additional statistically significant improvements in the evaluated traits. This finding should not be interpreted as demonstrating biological equivalence between the two doses or establishing an optimal supplementation level. Overall, the results support further evaluation of rumen-derived *S. macedonicus* as a probiotic candidate for growing lambs. Additional studies incorporating comprehensive antimicrobial-resistance characterization, genomic assessment, microbiome and metabolomic analyses, and longer-term safety evaluation are required before its safety and optimal inclusion level can be established.

## Figures and Tables

**Table 1:** Composition and nutrient levels (g/kg DM) of the basal diet that were tested in vitro and offered in vivo to the Barki lambs.

Item	Concentrate Mixture	Clover Hay	Control Diet
Composition (g/kg DM)			
Sugar beet pulp	300	-	180
Cottonseed meal	200	-	120
Yellow corn	200	-	120
Wheat bran	200	-	120
Soybean meal	50.0	-	30.0
Sodium bicarbonate	20.0	-	12.0
Limestone	15.0	-	9.00
Salt	10.0	-	6.00
MV mixture ^1^	5.00	-	3.00
Clover hay	-	1000	400
Nutrient levels (g/kg DM)			
DM (g/kg)	910.1	902.3	906.9
OM	930.5	923.1	927.5
Ash	69.50	76.90	72.46
CP	132.5	121.6	128.1
EE	65.30	41.10	55.62
NDF	370.6	460.1	406.4
ADF	159.2	249.3	195.2
NSC ^2^	362.1	300.3	337.4

^1^ Mineral and vitamin mixture (MV) which contained (per kg DM): 141 Ca, 87 g P, 45 g Mg, 14 g S, 120 g Na, 6 g K, 944 mg Fe, 1613 mg Zn, 484 mg Cu, 1748 mg Mn, 58 mg I, 51 mg Co, 13 mg Se, 248,000 U vitamin A, 74,000 UI vitamin D3, and 1656 IU vitamin E. ^2^ Non-structural carbohydrates.

**Table 2:** Antimicrobial activity response and antibiotic susceptibility of *Streptococcus macedonicus* against some pathogens and antibiotics using the disk diffusion method.

Pathogens	IZD (mm) ^1^
*E-coli*	18
*Salmonella typhimurium*	0
*Pseudomonas aeruginosa*	20
*Listeria monocytogenes*	0
*Yersinia enterocolitica*	0
*Bacillus cereus*	15
*Staphylococcus aureus*	0
Antibiotic (dose)	
Ampicillin (10 µg)	32
Penicillin (10 µg)	38
Vancomycin (30 µg)	13
Cefadroxil (30 µg)	28
Erythromycin (15 µg)	24
Streptomycin (10 µg)	4
Kanamycin (30 µg)	0
Tetracycline (30 µg)	28

^1^ Inhibition clear zone diameter (IZD) to the nearest whole mm.

**Table 3:** Effect of supplementing diet with different doses of *Streptococcus macedonicus* (SM) on the in vitro dry matter digestibility, gas, and methane production.

Item	Treatments ^1^					SEM	*p*
	Control	SM1	SM2	SM3	SM4		
pH	6.83	6.75	6.77	6.78	6.78	0.032	0.058
IVDMD, %	52.9 ^b^	53.4 ^ab^	54.7 ^a^	55.9 ^a^	56.7 ^a^	0.182	0.042
Ammonia-N, mM	11.3	11.9	11.6	11.3	11.1	0.055	0.088
TGP, mL	29.8 ^b^	46.3 ^a^	49.2 ^a^	49.0 ^a^	46.7 ^a^	1.236	<0.0001
TGP/g DM, mL	73.8 ^c^	115.4 ^b^	129.8 ^a^	122.4 ^a^	126.4 ^a^	2.652	<0.0001
CH4, mL	2.35 ^c^	3.46 ^a^	3.24 ^b^	3.21 ^b^	2.11 ^c^	0.012	0.003
CH4/g DM, mL	5.75 ^b^	8.62 ^a^	8.50 ^a^	7.97 ^a^	5.77 ^b^	0.111	0.004

^a–c^ Means within a row with different superscripts differ (*p* < 0.05). ^1^ Treatments: Control (60% of concentrate feed mixture + 40% of clover hay); SM1 (control diet + 0.002 g of *Streptococcus macedonicus* (SM)); SM2 (control diet + 0.004 g of SM); SM3 (control diet + 0.008 g of SM); SM4 (control diet + 0.016 g of SM).

**Table 4:** Effect of supplementing the diet of growing Barki lambs with two doses of *Streptococcus macedonicus* (SM) on feed intake and nutrient digestibility.

Item ^1^	Treatments ^2^				SEM	*p * ^3^		
	Control	CPro	SMD1	SMD2		Trt	SP	Trt × SP
DMI, g/d								
CFM	857.3 ^b^	893.2 ^a^	889.9 ^a^	885.1 ^a^	27.92	0.002	<0.001	0.092
CH	502.1 ^b^	553.7 ^a^	549.2 ^a^	542.8 ^a^	15.77	<0.001	<0.001	0.133
Total	1359.4 ^b^	1446.9 ^a^	1439.1 ^a^	1427.9 ^a^	39.22	<0.001	<0.001	0.451
Nutrient digestibility %								
DM	66.3 ^c^	71.1 ^b^	75.2 ^a^	74.3 ^a^	2.332	0.002	<0.001	0.823
OM	71.1 ^c^	75.9 ^b^	80.9 ^a^	79.5 ^a^	2.912	0.021	<0.001	0.511
CP	60.9 ^c^	65.7 ^b^	70.1 ^a^	68.8 ^a^	1.985	0.009	0.002	0.439
EE	54.8 ^b^	58.7 ^a^	59.3 ^a^	58.6 ^a^	1.566	0.001	0.033	0.123
NDF	58.6 ^c^	63.2 ^b^	65.5 ^a^	64.1 ^ab^	1.632	0.011	0.009	0.211
ADF	61.2 ^c^	65.3 ^b^	69.7 ^a^	69.3 ^a^	2.001	0.004	<0.001	0.235

^a–c^ Means within a row with different superscripts differ (*p* < 0.05). ^1^ Item: DMI, dry matter intake; CFM, concentrate feed mixture; CH, clover hay. ^2^ Treatments: Control (60% of concentrate feed mixture + 40% of clover hay); CPro (control diet + 1 g of commercial probiotic); SMD1 (control diet + 1 g of *Streptococcus macedonicus*); SMD2 (control diet + 2 g of *Streptococcus macedonicus*). ^3^ *p*-value: Trt (treatment); SP (sampling period).

**Table 5:** Effect of supplementing the diet of growing Barki lambs with two doses of *Streptococcus macedonicus* (SM) on the growth performance and feed conversion.

Item ^1^	Treatments ^2^				SEM	*p * ^3^		
	Control	CPro	SMD1	SMD2		Trt	SP	Trt × SP
IBW, kg	28.0	27.6	27.9	27.5	1.931	0.295	-	-
FBW, kg	50.1 ^c^	52.5 ^b^	56.8 ^a^	57.1 ^a^	3.122	0.002	-	-
WG, kg	22.1 ^c^	24.9 ^b^	28.9 ^a^	29.6 ^a^	1.162	0.011	-	-
ADG, g/d	105.2 ^c^	118.6 ^b^	137.6 ^a^	140.9 ^a^	5.356	<0.001	<0.001	0.111
FCR, g diet/g gain	12.9 ^a^	12.2 ^a^	10.5 ^b^	10.1 ^b^	0.122	<0.001	<0.001	0.135

^a–c^ Means within a row with different superscripts differ (*p* < 0.05). ^1^ Item: IBW, initial body weight; FBW, final body weight; WG, weight gain; ADG, average daily gain; FCR, feed conversion rate. ^2^ Treatments: Control (60% of concentrate feed mixture + 40% of clover hay); CPro (control diet + 1 g of commercial probiotic); SMD1 (control diet + 1 g of *Streptococcus macedonicus*); SMD2 (control diet + 2 g of *Streptococcus macedonicus*). ^3^ *p*-value: Trt (treatment); SP (sampling period).

**Table 6:** Effect of supplementing the diet of growing Barki lambs with two doses of *Streptococcus macedonicus* (SM) on the blood serum chemistry.

Item	Treatments ^1^				SEM	*p * ^2^		
	Control	CPro	SMD1	SMD2		Trt	SP	Trt × SP
Total proteins, g/dL	6.92	7.12	7.22	7.18	0.332	0.202	0.593	0.356
Albumin, g/dL	3.02	3.15	3.22	3.19	0.158	0.650	0.341	0.410
Globulin, g/dL	3.65	3.79	3.88	3.78	0.122	0.567	0.794	0.628
Glucose, mg/dL	62.9	64.2	65.1	63.9	3.992	0.185	0.257	0.726
Urea-N, mg/dL	45.6	47.1	46.5	46.9	2.123	0.678	0.249	0.266
Cholesterol, mg/dL	85.3	84.6	86.1	85.7	4.541	0.646	0.387	0.191
Triglycerides, mg/dL	59.3	61.2	60.9	59.1	2.874	0.216	0.197	0.738
GOT, Units/L	28.9	29.2	30.1	29.5	0.893	0.223	0.370	0.670
GPT, Units/L	18.9	19.2	19.8	20.2	1.102	0.404	0.419	0.164
T4, ng/mL	79.2	79.8	80.2	81.2	4.052	0.684	0.664	0.509

^1^ Treatments: Control (60% of concentrate feed mixture + 40% of clover hay); CPro (control diet + 1 g of commercial probiotic); SMD1 (control diet + 1 g of *Streptococcus macedonicus*); SMD2 (control diet + 2 g of *Streptococcus macedonicus*). ^2^ *p*-value: Trt (treatment); SP (sampling period). GPT: glutamate-pyruvate transaminase, GOT: glutamate-oxaloacetate transaminase, T4: thyroxine hormone.

**Table 7:** Effect of supplementing the diet of growing Barki lambs with two doses of *Streptococcus macedonicus* (SM) on the carcass traits and measurements.

Item	Treatments ^1^				SEM	*p*
	Control	CPro	SMD1	SMD2		
Live body weight, kg	51.6 ^c^	54.1 ^b^	58.6 ^a^	59.0 ^a^	2.569	<0.001
Hot carcass weight, kg	23.3 ^c^	25.9 ^b^	28.6 ^a^	29.3 ^a^	1.332	<0.001
Dressing, %	45.3 ^c^	47.9 ^b^	48.9 ^ab^	49.7 ^a^	1.698	0.002
Fat thickness, cm	0.36 ^a^	0.38 ^a^	0.33 ^b^	0.31 ^b^	0.032	0.018
Longissimus muscle area, cm^2^	19.1 ^c^	22.5 ^b^	25.8 ^a^	26.9 ^a^	1.589	<0.001
Lean, %	51.3 ^c^	54.3 ^b^	58.5 ^a^	57.9 ^a^	3.998	0.010
Fat, %	24.3 ^a^	22.3 ^b^	20.9 ^c^	20.2 ^c^	1.233	<0.001
Bone, %	22.4 ^a^	21.9 ^a^	20.6 ^b^	20.1 ^b^	1.002	0.001
Carcass measurements (cm)						
Carcass length	74.2 ^c^	76.1 ^b^	78.4 ^a^	79.3 ^a^	3.002	<0.001
Carcass height	53.3 ^c^	55.9 ^b^	59.1 ^a^	60.6 ^a^	2.922	0.001
Carcass depth	24.1 ^c^	30.2 ^b^	35.2 ^a^	36.1 ^a^	1.211	0.008
Head length	23.1	23.8	24.2	24.5	1.423	0.097
Head width	12.9	13.1	13.1	13.5	0.745	0.166
Paunch girth	89.2 ^c^	94.2 ^b^	98.5 ^a^	101 ^a^	3.882	0.001
Round width	16.5 ^c^	18.6 ^b^	20.3 ^a^	20.7 ^a^	0.898	0.002
Leg circumference	30.1 ^c^	33.9 ^b^	36.2 ^a^	37.4 ^a^	1.654	<0.001

^a–c^ Means within a row with different superscripts differ (*p* < 0.05). ^1^ Treatments: Control (60% of concentrate feed mixture + 40% of clover hay); CPro (control diet + 1 g of commercial probiotic); SMD1 (control diet + 1 g of *Streptococcus macedonicus*); SMD2 (control diet + 2 g of *Streptococcus macedonicus*).

**Table 8:** Effect of supplementing the diet of growing Barki lambs with two doses of *Streptococcus macedonicus* (SM) on the carcass physical and chemical characteristics.

Item	Treatments ^2^				SEM	*p*
	Control	CPro	SMD1	SMD2		
Chemical composition, %						
Moisture	72.9	73.3	73.1	72.7	3.921	0.069
Protein	20.9	21.2	21.8	21.8	1.005	0.087
Collagen	2.14	2.19	2.25	2.23	0.089	0.121
Fat	4.05	4.13	3.98	4.00	0.071	0.201
Physical traits						
WHC ^1^	74.9 ^c^	79.6 ^b^	85.9 ^a^	84.1 ^a^	3.147	0.002
Brightness	36.9	36.1	35.9	35.8	1.977	0.177
Redness	36.5 ^b^	37.9 ^b^	39.6 ^a^	40.1 ^a^	1.882	0.006
Yellowness	4.12	4.09	4.23	4.19	0.077	0.193
Chroma	19.2	19.9	20.1	20.0	0.823	0.174
Hue	12.9	13.3	12.5	13.9	0.412	0.097

^a–c^ Means within a row with different superscripts differ (*p* < 0.05). ^1^ WHC: water-holding capacity, ^2^ Treatments: Control (60% of concentrate feed mixture + 40% of clover hay); CPro (control diet + 1 g of commercial probiotic); SMD1 (control diet + 1 g of *Streptococcus macedonicus*); SMD2 (control diet + 2 g of *Streptococcus macedonicus*).

## Data Availability

The data presented in this study are available upon request from the corresponding authors.
